# *H. pylori *exploits and manipulates innate and adaptive immune cell signaling pathways to establish persistent infection

**DOI:** 10.1186/1478-811X-9-25

**Published:** 2011-11-01

**Authors:** Anne Müller, Mathias Oertli, Isabelle C Arnold

**Affiliations:** 1 Institute of Molecular Cancer Research, University of Zürich, Zürich, Switzerland; 2 Sir William Dunn School of Pathology, University of Oxford, Oxford, UK

**Keywords:** immune evasion, innate immune signaling, immunomodulation, persistent infection

## Abstract

Persistent infection with the gastric bacterial pathogen *Helicobacter pylori *causes gastritis and predisposes carriers to a high gastric cancer risk, but has also been linked to protection from allergic, chronic inflammatory and autoimmune diseases. In the course of tens of thousands of years of co-existence with its human host, *H. pylori *has evolved elaborate adaptations that allow it to persist in the hostile environment of the stomach in the face of a vigorous innate and adaptive immune response. For this review, we have identified several key immune cell types and signaling pathways that appear to be preferentially targeted by the bacteria to establish and maintain persistent infection. We explore the mechanisms that allow the bacteria to avoid detection by innate immune cells via their pattern recognition receptors, to escape T-cell mediated adaptive immunity, and to reprogram the immune system towards tolerance rather than immunity. The implications of the immunomodulatory properties of the bacteria for the prevention of allergic and auto-immune diseases in chronically infected individuals are also discussed.

## Innate immune receptor recognition of *H. pylori*

**Figure 1:**
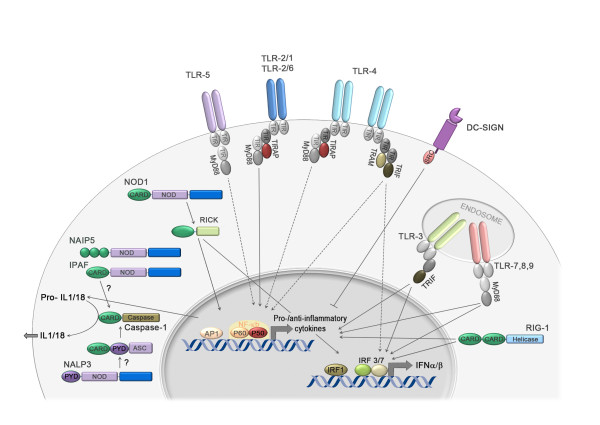
**Host innate immune receptors and signaling networks known or believed to participate in *H. pylori *recognition**. Toll-like receptors (TLRs), NOD-like receptors (NLRs), RIG-like receptors (RLRs), and C-type lectin receptors (CLRs) are expressed on the cell surface, in the endosomes, or in the cytosol of various types of immune cells. Their activation by specific pathogen-associated molecular patterns/ligands induces the expression of cytokine genes or results in the activation of caspase-1 and processing of IL-1β and IL-18. TLR2 and TLR9 recognize *H. pylori *LPS, lipoproteins and DNA, respectively [8-11]; in contrast, *H. pylori *LPS and flagellins are poor ligands of TLR4 and TLR5, respectively (indicated by dotted lines) [5,7]. The RIG-I-dependent recognition of *H. pylori *5'-triphosphorylated RNA in dendritic cells has been reported to induce expression of type I interferons [10]; fucosylated *H. pylori *ligands for the CLR DC-SIGN have been described, but appear to repress rather than activate signaling downstream of this surface-exposed CLR [12]. NOD1 recognizes *H. pylori *peptidoglycan and activates the transcription factors NF-κB and AP1 [16,17], and also triggers the production of type I interferons [21]. Comparatively little is known regarding additional possible NLR ligands (indicated by question marks). Conceivable, but unconfirmed *H. pylori *NLR ligands include flagellins (for NAIP5 and IPAF) and toxins (NALP3), which would activate the inflammasome and lead to the secretion of mature IL-1β and IL-18. Adapted from [1].

Innate immune cells as well as epithelial cells forming a first barrier to infection detect invading pathogens via their conserved microbial structures, the so-called pathogen-associated molecular patterns (PAMPs). Examples of PAMPs include microbial nucleid acids, and cell wall and flagellar components such as peptidoglycan, lipopolysaccharide (LPS), lipoproteins and flagellins [1]. PAMPs are recognized by at least four distinct classes of innate immune or pattern recognition receptors (PRRs) that are present either on cytoplasmic and endosomal membranes (Toll-like receptors, TLRs, and C-type lectin receptors, CLRs) or in the cytosol (NOD-like receptors, NLRs, RIG-like receptors, RLRs). Generally speaking, the ligation of TLRs, CLRs or RLRs results in the activation of pro-inflammatory transcription factors in the nucleus; in contrast, most NLRs are involved in the assembly of multi-protein complexes termed "inflammasomes", which process pro-IL-1β and pro-IL-18 to generate the mature, bioactive cytokines [2] (summarized in Figure 1).

PRRs are present not only on hematopoietic cells, but also on gastric epithelial cells which form a first line of defense against *H. pylori *infection [3,4]; therefore, many of the seminal studies in the field have focused on examining *H. pylori *recognition by epithelial cells. In particular, TLR4, 5 and 9 have been detected immunohistochemically on gastric epithelial cells in *H. pylori*-infected as well as uninfected patients, which in principle allows these cells to sense and respond to the infection [3,4]. Interestingly, *H. pylori *differs from other gastrointestinal pathogens in that it has evolved to largely avoid recognition by PAMPs. *H. pylori *flagellin is a poor ligand of TLR5 [5] due to mutations in the TLR5 recognition site of the N-terminal D1 domain of flagellin [6]. Indeed, mutating residues 89-96 of the strongly recognized *Salmonella *flagellin to the corresponding *H. pylori *flaA sequence abolishes TLR5 recognition [6]. The bacterium's LPS consists predominantly of the tetra-acylated lipid A variety, which is known to exhibit 1000-fold reduced bioactivity as compared to *E. coli *LPS [7]. *H. pylori *LPS activates the TLR4/MD-2 complex, but this has only been shown *in vitro *in cells overexpressing the complex [4]. Rather than being a strong TLR4 ligand, *H. pylori *LPS is thought to activate TLR2 on gastric epithelial cells, but again this has only been demonstrated *in vitro *using ectopic expression of the TLR [8,9]. Animal and cell culture experiments suggest that TLR2 ligands (LPS or other) indeed exist in *H. pylori *and related *Helicobacter *species [9-11], and can bind to TLR2 and activate NF-κB in epithelial cells [9]. However, the net effects of TLR2 ligation are anti-rather than pro-inflammatory *in vivo *as evidenced by lower *H. pylori *colonization levels and more severe immunopathology in gene-targeted mice lacking TLR2 [11]. While *H. pylori *DNA activates TLR9 expressed on dendritic cells (DCs) *in vitro *[10], TLR9^-/- ^mice do not differ from wild type mice in their ability to control *H. pylori *infections [11]. *H. pylori*'s 5'-triphosphorylated RNA can be sensed by the intracellular receptor RIG-1 and triggers type I interferons in DCs [10], but the role of this response in the control of the infection and the infection-associated pathology has to date not been explored. *H. pylori *further possesses fucosylated ligands for the C-type lectin receptor DC-SIGN [12]. However, in contrast to the mannosylated DC-SIGN ligands of *Mycobacterium tuberculosis*, which activate signaling downstream of DC-SIGN and trigger pro-inflammatory cytokine production, the DC-SIGN ligands of *H. pylori *actively dissociate the signaling complex downstream of this C-type lectin to suppress pro-inflammatory cytokine production [12]. The PRRs involved in sensing and responding to *H. pylori *are summarized in Figure 1. Taken together, the data available to date provide ample evidence of the impressive ability of *H. pylori *to avoid innate immune detection by the host's arsenal of PRRs, thereby preventing innate and adaptive immunity and ensuring its persistence.

## NOD1 signaling contributes to anti-*H. pylori *defense mechanisms

As mentioned earlier, the NLRs NOD1 and NOD2 recognize peptidoglycan metabolites of various pathogenic bacteria [13,14] and induce the transcription factor NF-κB to activate immune response genes [15]. Indeed, NOD1 was among the first PRRs shown to recognize an *H. pylori*-derived PAMP, i.e. its peptidoglycan [16], and has since attracted considerable attention. In their initial study, Ferrero and colleagues showed that peptidoglycan recognition by NOD1 was dependent on the expression of a functional Cag pathogenicity island (PAI)-encoded type IV secretion system (T4SS) and resulted in NF-κB activation [16]. More recently, the same group demonstrated that MAP kinases and the AP1 transcription factor were also activated upon peptidoglycan recognition by NOD1 [17]. NOD1 activation by *cag*PAI^+ ^*H. pylori *was further shown to be required for production of beta-defensins by gastric epithelial cells, which contributes to the bactericidal activity of cell culture supernatants against *H. pylori *[18]. *H. pylori *peptidoglycan delivery to cytosolic NOD1 via the T4SS occurs at cholesterol-rich microdomains referred to as lipid rafts, which contain high local concentrations of the T4SS receptor α5β1 integrin [19]; both cholesterol and α5β1 integrin are required for the T4SS-dependent delivery of peptidoglycan [19]. Although it was initially believed that peptidoglycan delivery occurs exclusively via the T4SS [16], Kaparakis *et al*. now provide evidence that outer membrane vesicles prepared from *cag*PAI^- ^*H. pylori *can also target peptidoglycan to cytosolic NOD1, and that intragastric delivery of peptidoglycan via outer membrane vesicles is sufficient to trigger innate and adaptive immune responses in mice [20].

**Figure 2:**
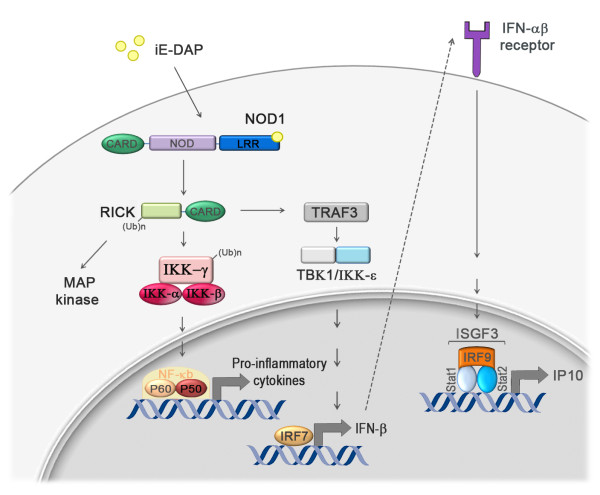
**Schematic of NOD1 signaling pathways**. NOD1 activation by its specific ligand iE-DAP activates at least two distinct signaling pathways. On the one hand, activated NOD1 triggers IFN-β production through the consecutive activation of RICK, TRAF3, TBK1/IKKε and IRF7. IRF7-dependent IFN-β production leads to the expression of IP-10 and other Th1 cytokines via transactivation of ISGF3 (Stat1-Stat2-IRF9 complex). On the other hand, NOD1 activation triggers the RICK and IKK-α,β,γ complex-dependent activation of NF-κB and subsequent production of proinflammatory cytokines and chemokines such as IL-8. Whether NF-κB is indeed activated upon Nod1 ligation in all cell types, including epithelial cells, remains controversial and will be the subject of future experimentation. Adapted from [56] and [21].

A novel pathway downstream of NOD1 was recently elucidated in an elegant study by Watanabe *et al*., who were able to link NOD1 signaling to the production of type I interferons and to the control of *H. pylori *infection [21]. Upon binding to its specific ligand, iE-DAP, NOD1 was shown to induce the sequential activation of the serine threonine kinase RICK, the TNF-associated factor 3 (TRAF3), the kinases TBK1 and IKKε, and ultimately the transcription factor IFN regulatory factor 7 (IRF7) [21]. IRF7 in turn induces IFN-β production, which leads to activation of the heterotrimeric transcription factor complex IFN-stimulated gene factor 3 (ISGF3) and the subsequent production of more IFN-β. Mice lacking the IFN-β receptor exhibit increased susceptibility to *H. pylori *infection, thereby phenocopying the defect of NOD1-deficient mice and suggesting that this pathway participates in host defenses against *H. pylori *[21]. The complete pathway is summarized in Figure 2. The study by Watanabe *et al*. refutes previous work showing that Nod1 activation induces NF-κB activity; at least in epithelial cells, the authors found no or minimal NF-κB activation upon Nod1 ligation by iE-DAP [21]. Another interesting recent study by Liu *et al*. showed that NOD1 and NOD2 are targets of the immunomodulatory glycoprotein olfactomedin 4 (OLFM4) in the context of *H. pylori *infection [22]. OLFM4 is an NF-κB target gene and associates directly with both NOD proteins, thereby creating a negative feedback loop that impairs *H. pylori*-induced NF-κB activation. OLFM4 knockout mice exhibited reduced *H. pylori *loads and enhanced gastric immune cell infiltration compared to wild type animals, suggesting that OLFM4 acts as negative regulator of *H. pylori*-specific, NOD-mediated immune responses [22].

## Inhibition of T-cell signaling and proliferation by the *H. pylori *virulence factors VacA and γ-glutamyl-transpeptidase

CD4^+ ^MHC class II-restricted T-cells are absolutely required for the control of experimental *H. pylori *infections and for the development of vaccine-induced protective immunity [23-25]. It therefore does not come as a big surprise that *H. pylori *has evolved virulence factors in the course of its 60.000+ years of co-existence with the human host [26] that allow it to suppress T-cell-mediated immunity. Seminal work by Haas, Cover, Baldari and their co-workers identified the vacuolating cytotoxin (VacA) as a key factor in the *H. pylori*-mediated inhibition of human T-cells [27-29]. VacA had initially been identified due to its ability to induce vacuolization of epithelial cells [30]. Haas, Cover, Baldari and colleagues extended these original findings to show that VacA inhibits cell proliferation by interfering with the T-cell receptor/interleukin-2 (IL-2) signaling pathway at the level of the Ca^2+^-calmodulin-dependent phosphatase calcineurin [27-29]. VacA thus prevents the nuclear translocation of the T-cell transcription factor NFAT, a global regulator of T-cell responses, resulting in downregulation of IL-2 gene transcription [27,28]. In subsequent studies, Haas and co-workers identified β2 (CD18) integrin to be the relevant receptor for VacA on human T-cells [31]. The authors were able to show that VacA exploits the recycling of lymphocyte function-associated antigen-1 (LFA-1; an integrin heterodimer on T-cells consisting of a β2 subunit associated with the CD11a α subunit) to become internalized by migrating human T-cells. LFA-1-deficient Jurkat T cells were resistant to vacuolation and IL-2 modulation, and genetic complementation restored their sensitivity to VacA [31]. A recent study further showed that VacA uptake depends on protein kinase C-mediated serine/threonine phosphorylation events, presumably of a specific threonine in the β2/CD18 cytoplasmic tail [32].

An alternative mechanism of T-cell suppression has been proposed by Gerhard and co-workers, who provided evidence in 2005 that human T-cell proliferation is blocked by *H. pylori *without accompanying effects on NFAT activation or cytokine production [33]. The authors postulated at the time that a secreted low-molecular-weight protein distinct from VacA arrests antigen-activated T-cells in the G1 phase of the cell cycle by interfering with G1 cyclin-dependent kinase activity [33]. The authors later demonstrated through a biochemical approach that the secreted γ**-**glutamyl transpeptidase (GGT) of *H. pylori *is the responsible factor for inhibition of T-cell proliferation; mutagenesis of GGT abrogated the inhibitory effect of the bacteria and recombinantly expressed GGT enzyme showed anti-proliferative activity [34]. The authors concluded from measuring reduced levels of c-Myc and phosphorylated c-Raf protein that GGT induces cell cycle arrest by disrupting the Ras signaling pathway [34].

## *H. pylori *infection induces "tolerogenic" DCs and regulatory T-cells with suppressive activity

Direct inhibition of T-cells by *H. pylori *virulence factors represents a compelling mechanism of immunosuppression and likely contributes to the bacteria's persistence in the human stomach. In addition, several laboratories have reported lately that *H. pylori*-specific effector T-cell responses are under the strict control of regulatory T-cells (Tregs) in infected humans, and that the depletion of Tregs improves immunological control over the infection and enhances vaccine-induced protective immunity in mouse models [35-38]. The degree to which the infected host generates *H. pylori*-specific Tregs appears to depend largely on the age at the time of infection [35]. Mice that are experimentally infected as adults with virulent *H. pylori *harboring the Cag pathogenicity island-encoded T4SS mentioned earlier rapidly develop gastritis and gastric cancer precursor lesions manifesting histologically as atrophic gastritis, intestinal metaplasia and epithelial hyperplasia [35]. In contrast, mice that are exposed to *H. pylori *during the neonatal period are protected against the development of such lesions, despite the fact that they are colonized at much higher levels than their adult-infected counterparts. The difference in *H. pylori *colonization and the associated pathology is closely linked to dramatic differences in local as well as systemic immune responses to the bacteria between the age groups. Neonatally infected mice develop immunological tolerance to the infection, which prevents them from generating *H. pylori*-specific T-helper responses and protects them from developing T-cell driven immunopathology. *H. pylori*-specific immunological tolerance depends on regulatory T-cells; their systemic depletion breaks tolerance and sensitizes mice to gastric immunopathology [35]. Whether immunological tolerance develops in humans infected early enough with *H. pylori *remains a matter of speculation. A study showing that infected children, but not adults, preferentially generate regulatory T-cell responses to the infection argues in favor of the tolerance model [39].

Above and beyond their role in preventing immunopathological gastric T-helper responses to the infection, Tregs are crucial mediators of the protection against allergen-induced asthma that is conferred by *H. pylori *infection in mice [40] and humans [41-44]. In infected mice subjected to an experimental protocol inducing airway inflammation and hyper-responsiveness through sensitization and challenge with ovalbumin, the protection against asthma can be abrogated by Treg depletion [40]. Conversely, asthma protection can be transferred from infected to uninfected mice by highly purified population of Tregs [40]. The exact mechanism of the suppression of *H. pylori*-specific or of allergen-specific effector T-cells by *H. pylori*-induced regulatory T-cells is not well understood; the secretion of suppressive Treg cytokines such as IL-10 and TGF-β, but also contact-dependent mechanisms of effector T-cell suppression can be envisioned. It is of interest to note in this context that *H. pylori *infection seems to be inversely correlated not only with asthma, but also with chronic inflammatory conditions of the small and large intestine. Several epidemiological studies have pointed out this association, looking at both ulcerative colitis and Crohn's disease patient populations [45,46]. Recent experimental evidence supports this concept; in a model of *Salmonella typhimurium*-induced colitis, concomitant *H. pylori *infection alleviated disease symptoms [47]. Protection against this form of colitis was attributed to reduced Th17 responses in the gut under conditions of co-infection, and coincided with the production of immunomodulatory (Treg-derived?) IL-10 in the mesenteric lymph nodes of the co-infected mice [47].

Inducible Tregs (iTregs; in contrast to "natural", thymus-derived nTregs) differentiate in the periphery as a result of their priming by dendritic cells (DCs) with "tolerogenic" rather than immunogenic properties [48]. Tolerogenic DCs convert naive T-cells into FoxP3^+ ^Tregs through antigen presentation in the absence of co-stimulatory signals or cytokines, either alone or in combination with the production of soluble and membrane-bound tolerogenic factors such as IL-10, TGFβ, retinoic acid, and programmed death ligands [48,49]. Tolerogenic properties have been attributed to immature DCs that have taken up antigen, but have not simultaneously been exposed to TLR or NLR ligands; such DCs are believed to acquire a semi-mature state characterized by high levels of MHCII, but low or no expression of co-stimulatory molecules or pro-inflammatory cytokines [48,49]. It is tempting to speculate based on the characteristic paucity of TLR and NLR ligands that is a hallmark of *H. pylori *that the pathogen has evolved precisely to avoid DC maturation, and to thereby promote Treg rather than T-effector responses. Experimental evidence for such Treg skewing comes from *in vitro *experiments with bone-marrow-derived DCs, which showed that *H. pylori*-experienced DCs appear to preferentially prime Treg over Th17 responses [50] and fail to produce pro-inflammatory cytokines [51]. The model of a predominantly tolerogenic role of DCs in the context of *H. pylori *infection is further corroborated by the demonstration that vaccine-induced protective immunity can be improved by systemic DC depletion [36] and by our finding that the lungs of neonatally infected mice subjected to experimental asthma induction are populated by semi-mature (and presumably tolerogenic) DCs [40].

## B-cells exhibit immunoregulatory rather than protective properties in the context of *H. pylori *infection

*H. pylori*-infected patients are characterized by high serum titers to *H. pylori *antigens, and serology is routinely used to diagnose the infection. Being a mucosal pathogen, *H. pylori *was initially suspected of triggering potentially protective mucosal IgA responses. However, early studies with mice lacking B-cells (μMT^-/- ^mice) or antibodies (IgA^-/-^) revealed that vaccine-induced protective immunity develops equally well in wild type and B-cell or antibody-deficient mice [24,52,53]. The results challenged the dogma that extracellular mucosal pathogens are controlled by antibodies and was confirmed again in a recent study introducing a novel and very effective new adjuvant derived from mycobacterial cell walls [36]. Subsequent work by Lycke and colleagues demonstrated that antibodies are not only dispensable for *H. pylori *clearance, but may even have detrimental effects on the human host [52]. μMT^-/- ^mice lacking B-cells altogether spontaneously reduce *H. pylori *burdens, but develop accelerated and aggravated gastritis [52]. Gene-targeting of the IL-10 and IgA-encoding genes synergizes to clear the infection, i.e. IL-10^-/-^/IgA^-/- ^mice clear *H. pylori *infections better than IL-10^-/- ^mice, both under conditions of experimental infection of naive as well as previously vaccinated mice [54]. The combined results show rather unequivocally that B-cells impair rather than promote immunity to *H. pylori*.

**Figure 3:**
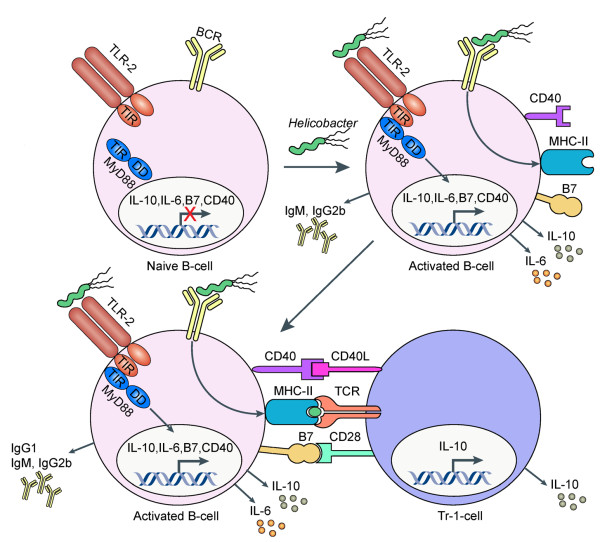
**TLR-2-activated B-cells suppress *Helicobacter*-induced preneoplastic gastric immunopathology by inducing T regulatory-1 cells**. Schematic representation of the events occurring in the course of *Helicobacter*-specific activation of B-cells at the site of infection (i.e. the gastric mucosa) and/or in the draining mesenteric lymph nodes. *Helicobacter *TLR-2 ligands activate B-cells in a MyD88-dependent manner, which leads to the expression and surface exposure of CD80, CD86 (together referred to as B7 molecules), the increased expression of CD40, and the secretion of IL-10, IL-6 and moderate amounts of TNF-α (the latter is not shown here) as well as antibodies of the IgM and IgG2b subclasses. The interaction of activated B-cells and naive T-cells induces T-cellular IL-10 expression and suppressive activity in a manner dependent on a direct interaction between both cell types via CD40/CD40L, B7/CD28 and MHCII/TCR. IL-10 secreting T-cells are essential players in the prevention of excessive *Helicobacter*-associated immunopathology. Adapted from [11].

We have recently attempted to elucidate the signaling pathways that play a role in B-cellular immunosuppression in the context of *H. pylori *infection. Interestingly, we found that B-cells exposed to *Helicobacter *extract produced large amounts of the regulatory cytokine IL-10 [11]. B-cell recognition of *Helicobacter *and the associated IL-10 production was entirely dependent on TLR2 and Myd88, as B-cells from the respective knock-out mice did not produce IL-10. *Helicobacter*-exposed B-cells further acquired the ability to efficiently induce IL-10 production in co-cultured, naive CD4^+ ^T-cells, thereby converting the T-cells to T-regulatory-1 (Tr1)-like cells with suppressive activity. *In vivo *studies using conditional knock-out mouse strains revealed that IL-10 production by T-cells, but not B-cells, was essential for the suppression of excessive gastric immunopathology. On the other hand, B-cells lacking Myd88 or TLR2 -in contrast to wild type B-cells- were incapable of preventing the characteristic infection-associated immunopathology of Myd88^-/- ^or IL-10^-/- ^strains when adoptively co-transferred with Tr1 cells [11]. Taken together, the results (summarized in Figure 3) suggest that the B-cell/Tr-1 cell axis is essential for balancing the control of *Helicobacter *infection with the prevention of excessive T-cell-driven gastric immunopathology.

## Conclusions

*H. pylori *has co-evolved with its human host for at least 30.000 years [55]. In contrast to most other bacterial pathogens, which temporarily cause virulent disease but are then rapidly cleared upon the onset of a pathogen-specific adaptive immune response, *H. pylori *persists in its host for decades, if not for life. This extraordinary ability to thrive in the face of a robust and vigorous local and systemic immune response is due to elaborate evolutionary adaptations of *H. pylori *that allow the bacteria to not only escape detection by pattern recognition receptors on innate immune cells, but to also evade adaptive immunity. The immunomodulatory properties of the pathogen reprogram the immune system towards immunological tolerance and assist the bacteria in establishing persistent infection. A byproduct of *H. pylori*-specific immunomodulation and immune tolerance is evident in Western societies that have largely eliminated the infection due to reduced transmission rates, frequent use of antibiotics in childhood and better sanitation. In these populations, the rates of asthma, allergies and other chronic inflammatory and auto-immune diseases have reached epidemic proportions; the inverse correlation of the incidence of such diseases and *H. pylor*i infection rates is striking and deserves further investigation. A better understanding of the signaling pathways and molecular players targeted by *H. pylori *to manipulate the host immune response and establish and maintain persistence will be instrumental for improving rational *H. pylori *vaccine design and possibly for exploiting *H. pylori*'s protective properties for asthma and allergy prevention and treatment.

## Competing interests

The authors declare that they have no competing interests.

## Authors' contributions

AM wrote the manuscript; ICA and MO contributed the figures. All authors have read and approved the final manuscript.
